# Validity of Weyl fermion picture for transition metals monopnictides TaAs, TaP, NbAs, and NbP from *ab initio* studies

**DOI:** 10.1038/s41598-018-21465-z

**Published:** 2018-02-23

**Authors:** Davide Grassano, Olivia Pulci, Adriano Mosca Conte, Friedhelm Bechstedt

**Affiliations:** 1 Dipartimento di Fisica, Università di Roma Tor Vergata, and INFN, Via della Ricerca Scientifica 1, I-00133 Rome, Italy; 2 Mediterranean Institute of Fundamental Physics (MIFP), Via Appia Nuova 31, I-00040 Marino Rome, Italy; 3 IFTO, Friedrich Schiller Universität, Max-Wien Platz 1, D-07743 Jena, Germany

## Abstract

We investigate electronic and optical properties of the topological Weyl semimetals TaAs, TaP, NbAs and NbP crystallizing in bct geometry by means of the *ab initio* density functional theory with spin-orbit interaction within the independent-particle approximation. The small energetical overlap of Ta5*d* or Nb4*d* derived conduction and valence bands leads to electron and/or hole pockets near the Fermi energy at the 24 Weyl nodes. The nodes give rise to two-(three-)dimensional Dirac cones for the *W*_1_ (*W*_2_) Weyl type. The band dispersion and occupation near the Weyl nodes determine the infrared optical properties. They are dominated by interband transitions, which lead to a deviation from the expected constant values of the imaginary part of the dielectric function. The resulting polarization anisotropy is also visible in the real part of the optical conductivity, whose lineshape deviates from the expected linearity. The details of the Weyl nodes are discussed in relation to recent ARPES results for TaAs and NbP, and compared with measured optical spectra for TaAs. The spectral features of the anisotropic and tilted Weyl fermions are restricted to low excitation energies above absorption onsets due to the band occupation.

## Introduction

Three-dimensional (3D) topological Dirac semimetals (TDSs) have been recently theoretically predicted, when including spin-orbit interaction (SOI), as a new topological state of 3D quantum matter^1^. They have attracted increasing attention in physics and materials science. The realization of such a new state has been proven for the semimetal phase of Cd_3_As_2_ and for the Na_3_Bi system by means of angle resolved photoemission spectroscopy (ARPES)^2,3^. A TDS can potentially be driven into other exotic phases with lower symmetry such as a topological Weyl semimetal (TWS)^4^. Whereas a trivial semimetal is characterized by small energy overlap between conduction and valence bands at different points in the Brillouin zone (BZ), a 3D TDS is defined in terms of linear bands in reciprocal space, which touch only at a set of isolated points in the BZ exactly at the Fermi energy *ε*_*F*_. They are a 3D generalization of graphene^5^, which can be identified as a 2D multivalley zero-gap semiconductor at two corner points of the BZ boundary. However, in contrast to graphene-like materials^6^ the Dirac points of such a TDS are not gapped by the spin-orbit interaction. Rather, the crossing of the linear bands at the Dirac points is protected by crystal symmetry^1^.

In the case of a 3D TDS both time-reversal (TR) and inversion (I) symmetry are present, resulting in four-component Dirac fermions near a Dirac point and doubly degenerate linear bands^5^. If either TR or I symmetry is broken, the two-fold degeneracy is lifted and only two linearly dispersing bands, that are degenerate at a Weyl point in the BZ, appear^7,8^. This degeneracy is robust against perturbations. If one Weyl node occurs at a point **k**_*W*_ in the BZ of a crystal without I symmetry, TR symmetry requires that another one occur at −**k**_*W*_. There must exist two more Weyl points at ${{\bf{k}}{\boldsymbol{^{\prime} }}}_{W}$ and −${{\bf{k}}{\boldsymbol{^{\prime} }}}_{W}$^1^. Pairs of Weyl nodes are formed at **k**_*W*_ and ${{\bf{k}}{\boldsymbol{^{\prime} }}}_{W}$ as well as −**k**_*W*_ and −${{\bf{k}}{\boldsymbol{^{\prime} }}}_{W}$. Under both TR and I symmetries, ${{\bf{k}}}_{W}={{\bf{k}}{\boldsymbol{^{\prime} }}}_{W}$ holds, four linear bands appear and the crystal becomes a TDS instead of a TWS. In such a picture Dirac fermions can be considered as two copies of Weyl fermions at the same BZ point. While in bulk systems the TWS is characterized by Weyl nodes near the Fermi level, non-closed Fermi arcs should be observable at surfaces of such crystals^4,9^.

Such Fermi-arc states have been predicted by *ab initio* calculations for TaAs^10^, and subsequently measured through ARPES^11,12^ for this and other materials such as NbP^13^. In the last few months there were several interesting studies of the extraordinary properties of transition metal monopnictides: direct observation of topological Fermi arcs for NbP, TaP, and TaAs by ARPES^10,13–17^, finding signatures of the Adler-Bell-Jackiw chiral anomaly in the magnetotransport of TaAs^18^, demonstration of helicity-protected ultrahigh mobility of Weyl fermions in NbP by magnetotransport measurements^19,20^, and finding of spectral features for linear bands in the measured optical conductivity^21^. More, interesting applications were suggested. They range from optical devices, Veselago lenses^22,23^, to Qubits^24^. These studies were supported or even driven by theoretical investigations of the electronic structure^10,12–15,25,26^. In addition, the static and dynamic stability^27^ as well as the spin polarization and quasiparticle interferences at surfaces^28,29^ were investigated.

Despite the recent progress, the complete picture and the chemical trends of Weyl fermions in transition metal monopnictides are still under debate. This concerns the details of the electron and hole pockets of the semimetals such as their positions in the energy-wave vector diagrams, i.e., the Weyl points, and the Fermi velocities in different directions. The energy range, in which Weyl fermions exist, and the role of anisotropy as well as tilting in the spectra, have to be clarified. The influence of the band structure of a TWS on its optical properties, especially the interplay of intraband and interband transitions, has to be studied and compared with the measured frequency dependencies. There are still notable differences in the electronic structure of e.g. NbP between ARPES measurements and first-principles calculations. Here, we present results of *ab initio* calculations for TaAs, TaP, NbAs, and NbP. Structural and electronic properties are presented. Special attention is paid to the consequences of asymmetry and tilting of the Dirac cones for the far-infrared optical absorption. Finally, a summary and conclusions are given.

## Results

### Atomic geometries

**Figure 1:**
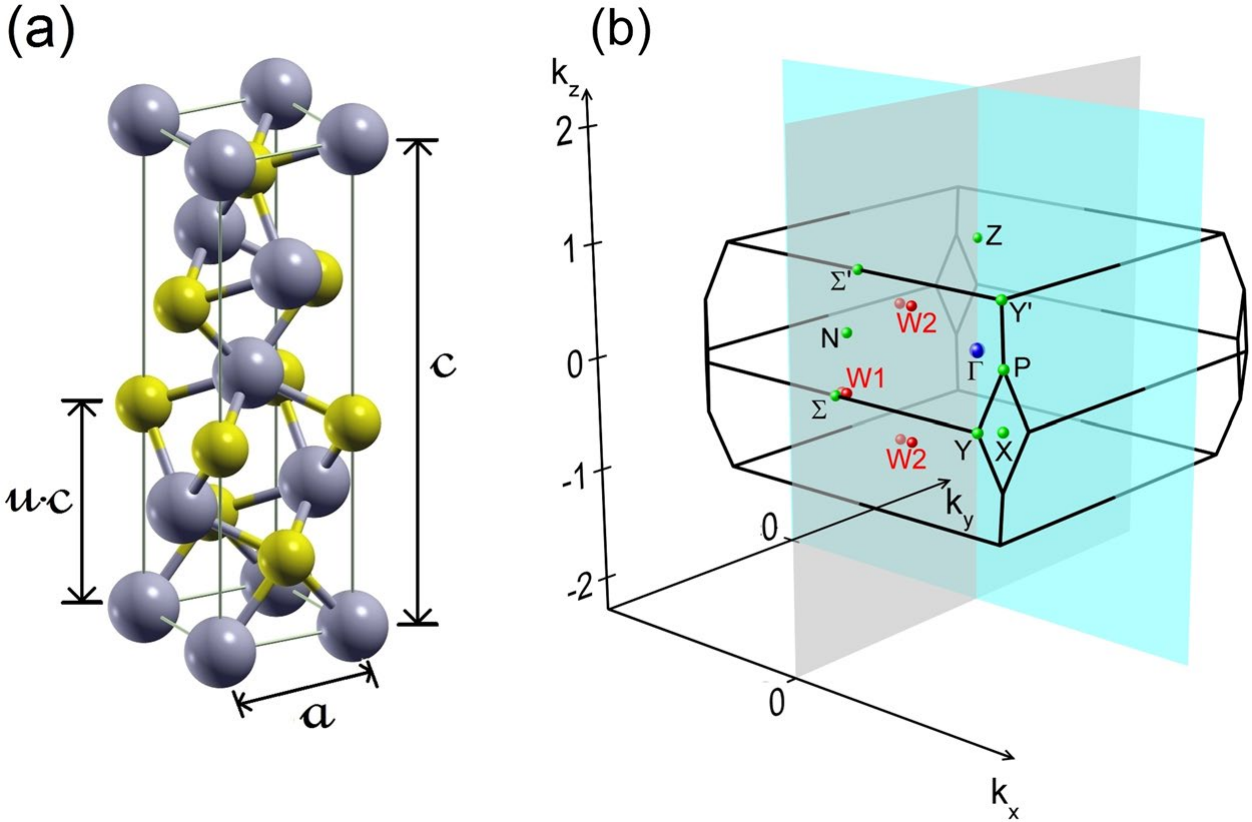
(**a**) Non-primitive unit cell (two times the primitive one) of a transition metal monopnictide crystallizing in a bct crystal structure with four cations (blue) and four anions (yellow). The two lattice constants *c* and *a* as well as the internal cell parameter *u* are indicated. (**b**) BZ of a bct crystal. Three examples for pairs of Weyl nodes are also displayed.

The considered class of transition metal monopnictides crystallizes in a body-centered tetragonal (bct) structure, whose non-primitive unit cell is displayed together with the BZ in Fig. 1. Its non-symmorphic space group I4_1_md (${{\rm{C}}}_{4v}^{1}$, no. 109) together with the atomic arrangement in Fig. 1a indicates lack of inversion symmetry, a fact being important for the occurrence of a Weyl semimetal. The multiplicity of the group is 4a, implying the presence of four atoms (two cations, two anions) in a primitive unit cell in the Wyckhoff positions (0, 0, *u*) and (0, 1/2, *u* + 1/4) with *u* = 0 for the Ta and Nb cations but finite *u* (see Table 1) for the As and P anions. The interpenetrating cation and anion sublattices are shifted by $(\frac{a}{2},\frac{a}{2},\delta )$ with *δ* ≈ *c*/12 against each other, as suggested by x-ray diffraction measurement for TaAs^30^ and confirmed here for all four monopnictides. The conventional cell in Fig. 1a illustrates that the crystals lack a horizontal mirror plane and thus inversion symmetry.

**Table 1:** Calculated lattice parameters *a*, *c*, and *u* of transition metal monopnictides.

Parameter	TaAs	TaP	NbAs	NbP
*a* (Å)	3.466 (3.437)	3.339 (3.318)	3.477 (3.452)	3.350 (3.334)
*c* (Å)	11.750 (11.656)	11.486 (11.363)	11.752 (11.679)	11.424 (11.376)
*u*	0.417 (0.417)	0.414 (0.416)	0.422 (0.417)	0.416 (0.416)
*B* (GPa)	84.38	102.31	87.47	95.99
*B*′	4.14	4.05	4.05	3.85
*E*_coh_ (eV/pair)	14.27	16.00	15.09	16.69

Experimental values (adapted from collection in^26^) are given in parenthesis. In addition, elastic and energetic parameters, the bulk modulus *B*, its pressure derivative *B*′, and the cohesive energy *E*_coh_, are listed.

During the lattice optimization we have also obtained the bulk modulus *B*, its pressure derivative *B*′, and the cohesive energy *E*_coh_ at the equilibrium coordinates. The *B* and *E*_coh_ values in Table 1 are of the same order but slightly lower than group-III (Al, Ga, In) phosphides and arsenides^31^. The large cohesive energies are in line with the experimental values of the elemental phases of Nb and Ta^32^, 7.57 and 8.10 eV/atom, respectively. The *B* and *B*′ parameters are typical for III-V compounds^33^. Calculations of *B* = 202.4 GPa for TaAs seems to lead to an overestimation due to the used hybrid XC functional^27^.

The results of our lattice optimization by means of the total energy are listed in Table 1 and compared with a collection of experimental values^26^. Thereby, the internal cell parameters *u* have been adapted to the above definition of Wyckhoff positions. The internal cell parameters *u* agree well with measured values and with the rule *u* ~ 5/12 found experimentally^30^. The calculated *a* and *c* lattice constants slightly overestimate the experimental ones by 0.4–0.8%. Only in the case of the *c* lattice constant of TaP the deviation is slightly larger with 1.1%. The reason for the general overestimation is the used PBE XC functional with gradient corrections^34^. There are also other experimental values *a* = 3.4348 Å and *c* = 11.641 Å for TaAs^30^, which are, also, close to our computed results.

### Band structures

**Figure 2:**
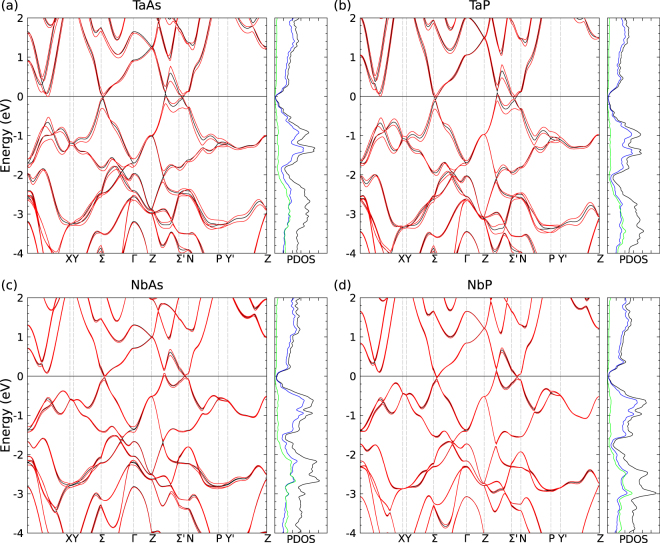
Band structure with SOI (red solid lines) and without SOI (black solid lines) of (**a**) TaAs, (**b**) TaP, (**c**) NbAs, and (**d**) NbP along high-symmetry lines in the bct BZ in Fig. 1b. The Fermi energy *ε*_*F*_ (black horizontal line) is chosen as energy zero. In addition, the DOS (black lines) and the orbital-symmetry-resolved DOS (Ta6*s* and Nb5*s*: red lines, Ta5*d* and Nb4*d*: blue lines, and As4*p* and P3*p*: green lines) are plotted.

The band structures of the four transition metal monopnictides TaAs, TaP, NbAs, and NbP are plotted in Fig. 2 along several high-symmetry lines in the bct BZ (see Fig. 1b). Since the bonds are highly ionic, the cations (anions) approach +3 (−3) charged ions, suggesting completely filled *p*^6^ shells of the anions and partially filled *d*^2^ shells of the cations, which build the upper valence bands. The orbital-resolved density of states (DOS) of the four compounds given also in Fig. 2 shows that Ta6*s* and Nb5*s* states are not visible in the presented energy range around the Fermi level.

The lowest conduction bands primarily consist of empty *d* states of the cations. The relatively shallow Ta5*d* and Nb4*d* shells also lead to broad valence bands which hybridize (in particular around 2.5 eV below the uppermost part of the occupied bands) with As4*p* and P3*p* orbitals, respectively. The lowest conduction and highest valence bands, which are mainly derived from cation *d* states, give rise to a small energetical overlap which leads to the formation of semimetals. Indeed, this tendency is visible for all four materials in Fig. 2 on the ΓΣ (near Σ), *Z*Σ′, and Σ′*N* lines. These regions in the four band structures are especially influenced by SOI, which lifts the double degeneracy of a band except at the Kramers points, which indicate the break of inversion symmetry but the respect of TR symmetry. The chemical trend is also obvious. The SOI influence decreases along the row TaAs, TaP, NbAs, and NbP in agreement with the reduction of the average atomic number.

Three regions exhibiting a small or vanishing gap and band crossings can be observed along the ΓΣ direction (near a *W*_1_ node), along the Σ′*N* and Σ′*Z* lines coming closer to trivial points. *W*_2_ nodes are not visible in Fig. 2 since they are not close to the zone boundaries (see Table 2).

**Table 2:** Coordinates of Weyl points *W*_1_, ${{\bf{k}}}_{{W}_{1}}$, and *W*_2_, ${{\bf{k}}}_{{W}_{2}}$, in *k* space together with their energy distance ${\varepsilon }_{{F}_{i}}$ to the Fermi level and resulting free carrier densities *n*_*i*_.

Weyl node	compound	*k*_*x*_ $(\tfrac{{\bf{2}}{\boldsymbol{\pi }}}{{\boldsymbol{a}}})$	*k*_*y*_ $(\tfrac{{\bf{2}}{\boldsymbol{\pi }}}{{\boldsymbol{a}}})$	*k*_*z*_ $(\tfrac{{\bf{2}}{\boldsymbol{\pi }}}{{\boldsymbol{c}}})$	${{\boldsymbol{\varepsilon }}}_{{{\boldsymbol{F}}}_{{\boldsymbol{i}}}}$ (eV)	*n*_*i*_ (10^17^ cm^−3^)
*W*_1_	TaAs	0.0078	0.5103	0	−0.026	1.07 (4.38)
	TaP	0.0077	0.5174	0	−0.055	15.00 (42.50)
	NbAs	0.0026	0.4859	0	−0.033	3.93 (31.21)
	NbP	0.0029	0.4921	0	−0.056	12.50 (380.75)
*W*_2_	TaAs	0.0198	0.2818	0.5905	−0.013	0.25 (0.05)
	TaP	0.0161	0.2741	0.5885	0.021	5.87 (0.26)
	NbAs	0.0064	0.2790	0.5736	0.004	0.53 (0.01)
	NbP	0.0047	0.2710	0.5770	0.026	3.78 (1.04)

The densities computed for purely linear bands (see eq. (4)) with the Fermi velocities of Table 3 are given in parenthesis.

The discussion of the behavior of the bands near the Fermi level, in particular, the crossing of conduction and valence bands, requires more detailed studies. The band structures in Fig. 2 display the C_4_ and mirror symmetries visible in real space in Fig. 1a. We study three Cartesian axes, 1 parallel to the tetragonal axis ([001]), and two perpendicular ([100] and [010]). Corresponding *k*_*z*_ and *k*_*x*_ as well as *k*_*y*_ directions are considered in **k** space.

With SOI each nodal line (see Fig. S1 in Supplementary Material) decays into six Weyl points, more precisely one pair of *W*_1_ nodes and two pairs of *W*_2_ nodes, which are displaced slightly from the mirror planes. Consequently, 24 Weyl points appear in total, 8 ones on the *k*_*z*_ = 0 plane, denoted with *W*_1_, and 16 Weyl points away from the *k*_*z*_ = 0 plane, called *W*_2_. The 24 points can be arranged in 12 pairs of opposite chirality^25^. For each transition metal monopnictide the positions of the Weyl points of classes *W*_1_ and *W*_2_ have been determined by generating a narrow grid of **k** points around the described regions, thereby focusing on band energies close to *ε*_*F*_. This sampling has been reiterated until the desired accuracy, namely the distance of conduction and valence bands <0.1 meV, was achieved. For one member of each *W*_1_ and *W*_2_ family the **k**-space location of the crossing Weyl point and its distance ${\varepsilon }_{{F}_{i}}$ (*i* = 1, 2) to the Fermi level *ε*_*F*_ are listed in Table 2. Only one set of *W*_1_ and *W*_2_coordinates is given in Table 2. The other **k** points can be obtained applying all possible mirror symmetry operations.

**Figure 3:**
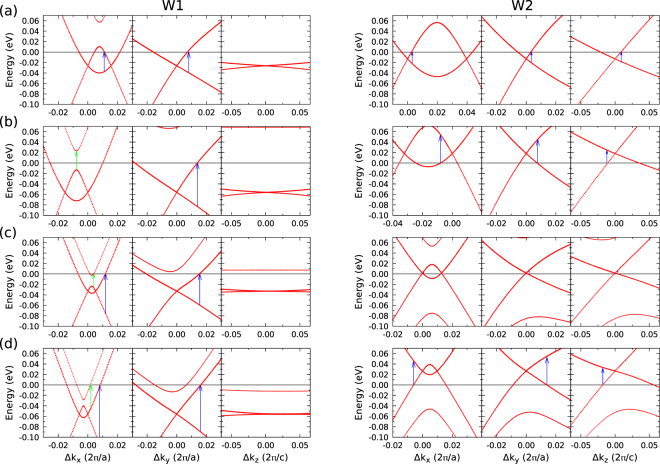
Bands (dotted  red lines) around the Fermi level in a small energy interval close to the Weyl nodes in Table 2 in *k*_*x*_, *k*_*y*_, and *k*_*z*_ direction. The **k**-space location of the Weyl nodes is used as coordinate zero. The zero energy gives the Fermi level (horizontal black line). In the *k*_*x*_ case, pairs of Weyl nodes are displayed. The vertical arrows indicate possible onsets of the interband transitions in the vicinity of Weyl nodes. Blue arrows: transitions between Weyl bands; green arrows: transitions between Weyl and non-topological bands.

The ${\varepsilon }_{{F}_{i}}$ values in Table 2 are small, of the order of a few meV. The absolute values are mainly determined by the As or P anion. In the average, the energy offsets between the two nodes are about 25 meV for arsenides and 79 meV for phosphides, respectively. The ${\varepsilon }_{{F}_{i}}$ values are in excellent agreement with other DFT calculations using the GGA-PBE XC functional despite using different codes and slightly different atomic coordinates^14,15^. This is not only true for the **k**-space locations ${{\bf{k}}}_{{W}_{1}}$ and ${{\bf{k}}}_{{W}_{2}}$ but also for the energy differences ${\varepsilon }_{{F}_{i}}$. The maximum deviations are less than 4 meV. Good agreement is also found with data of ref.^25^ if one takes into consideration that other but symmetry-equivalent Weyl nodes have been chosen. The results in Table 2 also agree well with experimental findings. For instance, magnetotransport measurements on NbP indicate *W*_1_ points 57 meV below *ε*_*F*_, while *W*_2_ ones are 5 meV above *ε*_*F*_^19^. The first energy is in complete agreement with Table 2. Only in the *W*_2_ case we find a slightly larger value. ARPES measurements of TaAs show that the native chemical potential is very close to the energy of the *W*_2_ Weyl nodes as in Fig. 3a and Table 2 ^18^. For TaP it is estimated to be about 24 meV below the *W*_2_ nodes^15^. For TaP, NbAs, and NbP our data in Table 2 reveal the coexistence of hole- and electron-type TWS pockets in the *k*_*z*_ = 0 plane (*W*_1_) and in the *k*_*y*_ = 0 and *k*_*x*_ = 0 plane (*W*_2_), respectively. This is experimentally confirmed for NbP by magnetoresistance measurements^20^ and ARPES^16^. The measurement of the Fermi arcs by ARPES is more difficult for NbP^16^. The smaller SOI splitting decreases the separation of Weyl points compared to TaAs. The signs of ${\varepsilon }_{{F}_{i}}$ in Table 2 show that the *W*_1_ Weyl nodes give rise to eight electron pockets, while *W*_2_ nodes are related to 16 hole pockets in the BZ. That means that the transition metal monopnictides possess a rather complex Fermi surface. TaAs represents an exception, because electron pockets also occur at *W*_2_ nodes. Consequently, TaAs is not really a semimetal, rather a metal but with only a very low density of the conduction electrons.

The *ab initio* determination of the electron and hole densities from the computed bands is extremely difficult (see illustration in Fig. 3), because, in general, only a small surroundings of a Weyl node in **k** space contributes. Additionally, the closeness of the bands within one pair of Weyl nodes and the extremely flat *W*_1_ bands parallel to the tetragonal axis (see Fig. 3) give rise to complications. The band linearity is restricted to small surroundings of **k**_*W*_ in reciprocal space. As a consequence, the Weyl fermion picture is limited to small energy intervals of the order of a few 10 meV. The accuracy of our computed values has been increased by using an extremely dense **k**-point meshes around the Weyl nodes characterized by a cutoff of 0.08 × 2*π*/*a* or 2*π*/*c*. The complex band structures between two Weyl nodes of a pair and the flat bands around *W*_1_ points in tetragonal-axis direction as well as the resulting numerical uncertainties may be responsible for deviations from other calculations. Apart from TaAs the large hole densities for TaP, NbAs, and NbP computed in ref.^26^ cannot be confirmed by our estimates using high **k** point densities around the Weyl nodes. The agreement is also difficult with densities extracted from experimental data. For NbP we estimate an electron density 0.1 × 10^19^ cm^−3^ by means of *the ab initio* method which is smaller than the value 2.5 × 10^19^ cm^−3^ derived within the effective-mass approximation (EMA) for both electrons and holes extracted from magnetotransport data^19^. Our band calculations (see Fig. 3 and its discussion), however, demonstrate that the EMA is hardly applicable. The extraction of carriers densities around Weyl nodes within the EMA from Hall measurements seems to tend to distinctly larger densities. Moreover, the carriers densities do not only depend on the method of their determination but also on the real structure of the samples. In addition, the presence of trivial hole pockets in the topological monopnictides may have strong influence on the measured carrier densities with an increasing tendency going from TaAs to NbP.

### Weyl fermions

In Weyl semimetals of the transition metal monopnictide class the energies of the Weyl nodes in Table 2 differ only by a few meV, between 4 and 56 meV, from the Fermi level position. Consequently, the physical properties of the corresponding Weyl fermions should be easily accessible after weak electron or hole doping of the system, which slightly shifts the Fermi energy. For instance, a minor p-doping may bring TaAs close to a system without free carriers as Dirac semimetals. For a better characterization of the linearly dispersive character we investigate the bands more carefully near the Weyl nodes. They are displayed in Fig. 3 for TaAs, TaP, NbAs, and NbP including SOI for a small energy interval around *ε*_*F*_ and small variations of the **k** vector around the **k**-space location of the *W*_1_ and *W*_2_ Weyl points in Table 2. As shown in Fig. 1b pairs of *W*_1_ and *W*_2_ Weyl nodes are extremely close to each other. The neighboring Weyl node in a Weyl pair is displaced, in *k*_*x*_-direction, by 0.016 (TaAs, TaP), 0.005 (NbAs), and 0.006 (NbP) in the *W*_1_ case, and 0.039 (TaAs), 0.032 (TaP), 0.012 (NbAs), and 0.009 (NbP) in the *W*_2_ case (in units of 2*π*/*a*). The close pair positions in the BZ, especially for Nb compounds, are the reason why the linearity of the bands and the occupation are significantly modified along the connection lines between the nodes in a pair. Nevertheless, all the 24 panels in Fig. 3 show that the bands in a near (with respect to energy and **k** variation) vicinity around the Weyl nodes can be linearized, but form tilted asymmetric Dirac cones. Only in the small vicinities around the nodes the Weyl character of the single particle excitations is conserved. Moreover, the band dispersion along *k*_*z*_ in the proximity of the *W*_1_ node almost vanishes, leading to an electron behaviour akin to that of a 2D system. This surprising finding should be detectable in transport measurements. The effect of asymmetry, tilting, and small energies of Weyl fermions will be discussed below in detail.

The dispersion of occupied bands as well as the relative positions to the Fermi level as displayed in Fig. 3 have been confirmed experimentally for TaAs and TaP^14,15^. Indeed, the complex behavior of the bands between the pairs of *W*_1_ and *W*_2_ Weyl nodes as illustrated in the left panels in Fig. 3 has been also observed experimentally. For the *W*_2_ nodes of TaAs and TaP the significant anisotropy of the linear bands along +*k*_*z*_- and −*k*_*z*_-directions as well as the position of 13 meV below or 21 meV above the Fermi level (see Table 2) are also confirmed by ARPES spectra measurements^14,15^. The same holds for the *W*_1_ nodes, both below the Fermi level. For a more extended energy interval (up to −1 eV) such bands around pairs of *W*_2_ nodes have been observed. Typically, below hole energies of about 0.2 eV deviations from their linearity is visible also in the measured dispersions. The distance between two *W*_2_ nodes along the *k*_*z*_-direction of about 1.19 $\frac{2\pi }{c}$ (see Table 2) for TaAs and TaP also agree with the experimental findings. Along the *k*_*x*_-direction adjacent *W*_1_ Weyl nodes with opposite chirality have been detected for TaAs^14^ (there identified with the *y* direction). The observed less asymmetric Dirac cones along *k*_*x*_ (in Fig. 3 along *k*_*y*_) agree qualitatively. However, direct comparison of bulk bands is difficult because the Weyl nodes are usually not located at high-symmetry lines in the BZ. The different chiralities of the Weyl fermions in the two paired nodes along *k*_*x*_ in Fig. 3 are obvious because of the opposite asymmetry and tilting along the positive and negative *k*_*x*_ directions.

Near the band crossings the low-energy bands can be linearized in the variation ${\rm{\Delta }}{\bf{k}}={\bf{k}}-{{\bf{k}}}_{{W}_{i}}$ of the **k** vector with respect to the position ${{\bf{k}}}_{{W}_{i}}$ of the Weyl node in Table 2. The prefactors are *ħ* multiplied with Fermi velocity *v*_*F*±*j*_. To fit the linearities of the bands in Fig. 3, for each Cartesian direction *j* = *x*, *y*, *z* we write

1 $${\varepsilon }_{\pm }({k}_{{W}_{i}j}+{\rm{\Delta }}{k}_{j})=\pm \hslash {v}_{F\pm j}{\rm{\Delta }}{k}_{j}$$

at each Weyl point of class *i* = 1, 2. In positive (+) Δ*k*_*x*_, Δ*k*_*y*_ or Δ*k*_*z*_ direction a positive slope and, hence, a positive Fermi velocity is derived. In the negative (−) directions the Fermi velocities are therefore chosen to be negative. The results of the fit procedure are listed in Table 3. Together with Fig. 3 they, indeed, confirm the three-dimensional and almost two-dimensional linear band features in the vicinity of Weyl nodes. However, the Fermi velocities in Table 3 indicate highly anisotropic and tilted 3D Dirac cones. On the other hand, the order of magnitude, a few 10^5^ m/s, is typical for Fermi velocities in 3D but also 2D Dirac cones^6,35^. The strong anisotropy of the Fermi velocities in *k*_*x*_- and *k*_*y*_-directions is a consequence of the asymmetric **k**-space location of the *W*_1_ and *W*_2_ Weyl nodes with respect to these coordinates. The equivalence of the *x*- and *y*-directions in a bct crystal is only visible for physical observables, not for isolated Weyl points. Comparing our results with Fermi velocities derived from other calculations^26^ we observe the same signs, order of magnitude, and chemical trends. Only the asymmetry (tilting) of the Dirac cones along a Cartesian axis is reduced compared with the values in ref.^26^, in particular for TaAs but less for NbP. As mentioned above, a surprising observation from all calculations is the almost vanishing Fermi velocities in *k*_*z*_-direction for *W*_1_ nodes, indicating nearly 2D Dirac cones along this direction.

**Table 3:** Fermi velocities of the bands crossing at *W*_1_ and *W*_2_ Weyl nodes in direction *k*_*x*_, *k*_*y*_, and *k*_*z*_, respectively.

Fermi velocities	*W*_1_				*W*_2_			
	TaAs	TaP	NbAs	NbP	TaAs	TaP	NbAs	NbP
*v*_*F*+*x*_	3.2	3.7	3.0	3.0	2.6	2.0	2.5	1.7
*v*_*F*−*x*_	−5.3	−5.4	−4.8	−5.1	−4.3	−3.9	−3.2	−2.4
*v*_*F*+*y*_	3.2	3.6	2.1	2.9	3.5	3.1	2.3	2.0
*v*_*F*−*y*_	−1.4	−1.5	−1.4	−1.6	−1.8	−2.1	−1.3	−1.7
*v*_*F*+*z*_	0.2	0.2	0.1	0.0 (3)	4.4	4.2	3.6	4.2
*v*_*F*−*z*_	−0.2	−0.2	−0.1	−0.0 (3)	−1.6	−1.4	−1.2	−1.4

All values are in 10^5^ m/s.

### Optical spectra

#### General frequency dependence

**Figure 4:**
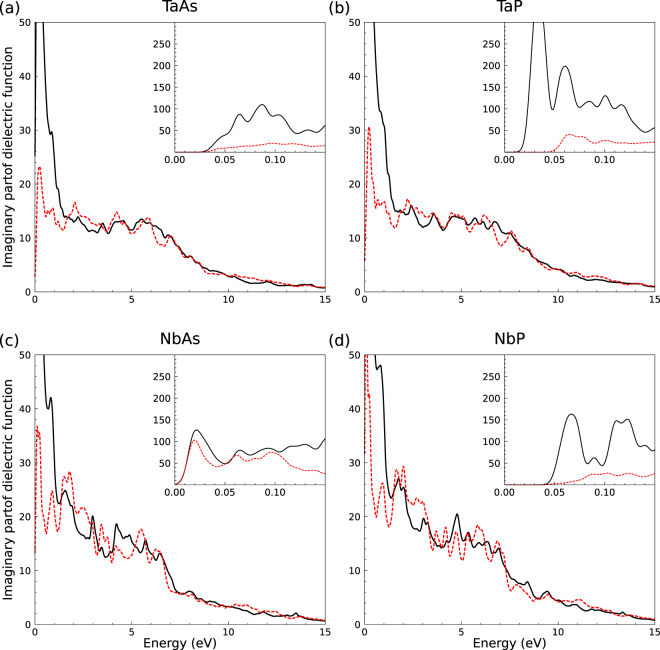
Imaginary part of the dielectric function of (**a**) TaAs, (**b**) TaP, (**c**) NbAs, and (**d**) NbP for light polarization perpendicular (black solid lines) and parallel (red dashed lines) to the tetragonal axis. The lorentzian broadening parameter used is 0.05 eV. The insets (calculated with a higher **k**-point density and a lorentzian broadening of 0.01 eV) show the asymptotic behavior for *ω* → 0.

**Figure 5:**
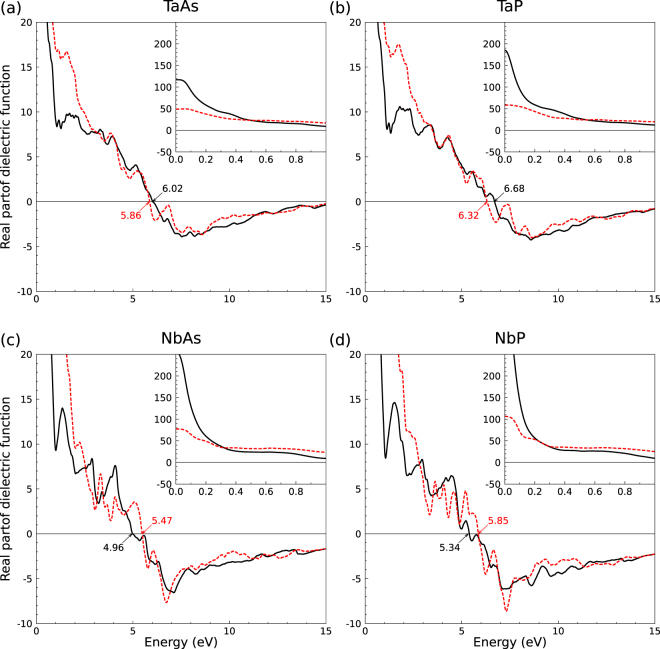
Real part of the dielectric function. Same denotations as in Fig. 4.

The convergence of the optical spectra with the number of bands and **k** points in the BZ has been carefully tested. For the interband case (10) we found that, including SOI, 80 empty bands are sufficient to obtain converged spectra up to a photon energy *ħω* = 30 eV. The BZ sampling is done with a 60 × 60 × 20 Monkhorst-Pack grid to account for the optical interband transitions in the entire BZ. A lifetime broadening parameter Γ = 0.05 eV is applied. The corresponding spectra, imaginary and real part of the interband dielectric function, are displayed in Figs 4 and 5 for TaAs, TaP, NbAs, and NbP. The insets in the panels for the imaginary parts, however, have been computed with a more dense **k**-point grid of 192 × 192 × 64, in order to sample the vicinities of the 24 Weyl nodes more precisely. Together with the trivial points they dominate the optics in the far infrared region.

The tetragonal symmetry of the four crystals is represented by different curves Im*ε*_*jj*_(*ω*) in Fig. 4 for light polarization perpendicular (black solid lines) and parallel (red dashed lines) to the tetragonal *z* axis. The imaginary parts strongly increase to large values for small photon energies. However, for *ħω* → +0, the insets of Fig. 4 show a vanishing imaginary part. This onset is a consequence of the band occupations near the Weyl points, which simulate gap-like onsets in the optical absorption. This behavior is different from a Dirac semimetal where the Fermi level crosses the Dirac point^35^. The plateaus in the Im*ε*_*jj*_(*ω*) spectra ruled by the fine structure constant and the Fermi velocities disappear in TWS spectra (Fig. 4). However, the optical anisotropy remains strong in the low-energy region of the imaginary parts.

For light polarization perpendicular to the *z* axis the optical absorption is much higher than that for parallel polarization. For *ħω* → ∞ the spectra show the typical *ω*^−2^ decay and a reduction of the polarization anisotropy.

The real parts of the interband dielectric function in Fig. 5 strongly increase their values in the infrared region. The detailed spectral features are determined by the band occupation near the Weyl points. While for *z* polarization the *ω* → 0 values tend to large interband contributions of the order of Im*ε*_*jj*_(0) = 50 (TaAs, TaP) or 150 (NbAs, NbP), for perpendicular polarization the *ε*_*jj*_(0) values approach to much large values. Exact data are difficult to give because of the finite **k**-point sampling and the used broadening parameter. In any case, the optical anisotropy is extremely pronounced in the low-energy region. For increasing photon energies the two curves *Reε*_*jj*_(*ω*) in the four panels of Fig. 5 show the characteristic behavior of interband dielectric functions.

#### Pure Weyl fermion picture

For a better understanding of the frequency behavior Im*ε*_*jj*_(*ω* → 0) (see insets in Fig. 4), we approximate the electronic structure by almost linear bands near the Weyl points. As a generalization of the fit eq. (1) we apply a three-dimensional expression for linear dispersion

2 $${\varepsilon }_{\pm }({\bf{k}})=\pm \hslash {\{\sum _{j=x,y,z}{[{v}_{F\pm j}{({\bf{k}}-{{\bf{k}}}_{W})}_{j}]}^{2}\}}^{\frac{1}{2}}$$

with + (−) the band above (below) a Weyl node at **k**_*W*_.

At energy *ε* = 0, the corresponding density of states can be analytically described using the DOS expression after eq. (12) and averaged Fermi velocities in *x*- and *y*-direction. It holds for *W*_*i*_ (*i* = 1, 2)

3 $$\begin{array}{rcl}{D}_{{W}_{i}}(\varepsilon ) & = & \frac{1}{2{\pi }^{2}{\hslash }^{3}{v}_{{F}_{x}}^{i}{v}_{{F}_{y}}^{i}{v}_{{F}_{z}}^{i}}{\varepsilon }^{2},\\ {v}_{{F}_{j}}^{i} & = & \frac{1}{2}({v}_{F+j}^{i}-{v}_{F-j}^{i})\quad (j=x,y\mathrm{).}\end{array}$$

This approximate treatment of the linear dispersions in *k*_*x*_- and *k*_*y*_-directions also allows to estimate the electron or hole density *n*_*i*_ at a Weyl node *W*_*i*_ as

4 $${n}_{i}=\frac{1}{6{\pi }^{2}{\hslash }^{3}{v}_{{F}_{x}}^{i}{v}_{{F}_{y}}^{i}{v}_{{F}_{z}}^{i}}{|{\varepsilon }_{{F}_{i}}|}^{3}\mathrm{.}$$

The corresponding results obtained using the Fermi velocities in Table 3 are listed in Table 2 in parenthesis. Because of the fact that for *W*_1_ the bands are extremely flat in *k*_*z*_-direction, the carrier densities (4) are significantly overestimated for the *W*_1_ nodes, especially for the Nb monopnictides, compared to the densities computed *ab initio*. The strong deviations are mainly a consequence that in (3) the $({\bf{k}}-{{\bf{k}}}_{{W}_{i}})$ integration has been extended to infinite, although it should be limited to small values.

The corresponding optical interband matrix elements in (10) for the transitions between tilted upper and lower cones can be approximated by (*j* = *x*, *y*, *z*)

5 $${|\langle +{\bf{k}}|{p}_{j}|-{\bf{k}}\rangle |}^{2}={m}^{2}\frac{1}{2}({v}_{F+j}^{2}+{v}_{F-j}^{2})\frac{{({\bf{k}}-{{\bf{k}}}_{{W}_{i}})}_{j}^{2}}{|{\bf{k}}-{{\bf{k}}}_{{W}_{i}}{|}^{2}}{F}_{i}$$

for wave vectors **k** in the vicinity of a Weyl point ${{\bf{k}}}_{{W}_{i}}$. The average over the left and right Fermi velocities guarantees the correct macroscopic symmetries of the sum over all *W*_1_ and *W*_2_ nodes. Such a direct relation (5) between momentum matrix elements and Fermi velocities has been proven for 2D and 3D graphene-like materials such as silicene, germanene, and Cd_3_As_2_^35,36^. In order to account for the chirality of the band states and the tilting of the cones a factor *F*_*i*_ < 1 has been introduced in (5) as a modification of the expression for graphene-like materials with isotropic Fermi velocity, where *F*_*i*_ = 1 holds.

The **k**-integration around the Weyl nodes in (10) can be approximately carried out for the linear bands (2), if the **k** dependence of the offset for the interband transitions is treated in an effective manner. The difference ${\varepsilon }_{{F}_{i}}-{\varepsilon }_{{F}_{i}}^{\ast }$ accounts for the asymmetry, especially the tilt, of the Dirac cones. In the limit of symmetric cones it holds ${\varepsilon }_{{F}_{i}}={\varepsilon }_{{F}_{i}}^{\ast }$. The problem can be well illustrated for the *W*_1_ nodes of TaAs. Figure 3a shows the lowest possible interband transition energie below 30 meV, while 2${\varepsilon }_{{F}_{1}}$ = 52 meV (see Table 2). For the 24 Weyl points of type *W*_1_ and *W*_2_ one finds, with *w*_1_ = 8 and *w*_2_ = 16,

6 $${\rm{Im}}\,{\varepsilon }_{jj}(\omega )=\frac{\alpha }{12}\,\sum _{i=1,2}\,{w}_{i}{F}_{i}\theta (\hslash \omega -\mathrm{2|}{\varepsilon }_{{F}_{i}}^{\ast }|)\frac{c}{{\bar{v}}_{{F}_{j}}^{i}}$$

with the Sommerfeld finestructure constant *α* = *e*^2^/*ħc* and the effective onset energy ${\varepsilon }_{{F}_{i}}^{\ast }$ for a Weyl point *W*_*i*_ (*i* = 1, 2) measured with respect to the node energy. Thereby, an approximated average Fermi velocity ${\bar{v}}_{{F}_{j}}^{i}$ for the polarization direction *j* and the Weyl node *W*_*i*_ has been introduced according to

7 $$\begin{array}{rcl}\frac{1}{{\bar{v}}_{{F}_{j}}^{i}} & = & \frac{3}{{v}_{{F}_{z}}^{i}}\{({\delta }_{jx}+{\delta }_{jy})\,\frac{1}{2}\,[1-\frac{1}{{\gamma }^{2}}(1-\frac{1}{\gamma }\,\arctan \,\gamma )]+[{\delta }_{jz}\frac{1}{{\gamma }^{2}}(1-\frac{1}{\gamma }\,\arctan \,\gamma )]\},\\ \gamma  & = & \sqrt{{(\frac{{v}_{{F}_{x,y}}^{i}}{{v}_{{F}_{z}}^{i}})}^{2}-1},\quad {v}_{{F}_{x,y}}^{i}=\frac{1}{4}\,\sum _{j=x,y}\,({v}_{F+j}^{i}-{v}_{F-j}^{i}),\end{array}$$

where an average Fermi velocity ${v}_{{F}_{x,y}}^{i}$ in the *xy*-plane perpendicular to the tetragonal axis has been defined to represent expression (6) in terms of the abbreviation (7). In principle, its introduction is in line with the macroscopic tetragonal symmetry, which has to be fulfilled for the dielectric function but not for the contribution of a single Weyl node. Nevertheless, the velocity ${\bar{v}}_{{F}_{j}}^{i}$ still accounts for the strong deviations from the ideal Dirac cones by the anisotropy, tilting, and violation of symmetry between upper and lower cones in the case of the TWSs, despite the symmetrization used for the *k*_*x*_- and *k*_*y*_-directions. This is clearly described by the different expressions parallel or perpendicular to the tetragonal axis and the strong deformation of the Dirac cones expressed by the parameter *γ*. The strong anisotropy of the *W*_1_ Weyl nodes results in values $\gamma \gg 1$, whereas *γ* vanishes or has a small imaginary value for *W*_2_ nodes.

The combination of the formulas (6) and (7) for *F*_*i*_ ≡ 1 and studying the limits, *ω* → +0 and ${\varepsilon }_{{F}_{i}}\to 0$, allows to extend to the case of a Dirac semimetal-like situation with an almost constant plateau in the imaginary parts. The limits, which can be realized in a slightly doped TaAs, lead to approximate values Im*ε*_*zz*_(0) = 12, 10, 11, and 8 as well as Im*ε*_*xx*_(0) = Im*ε*_*yy*_(0) = 112, 79, 451, and 145 for TaAs, TaP, NbAs, and NbP, respectively. This trend is not visible in Fig. 4, where for low energies Im*ε*_*jj*_(0) = 0 due to the occupation of the Weyl nodes. Moreover, the velocity fit in the case of flat *W*_1_ bands along *k*_*z*_ introduces quite a large error. The effect of the almost 2D Dirac cones in the *W*_1_ case is not described as well by the averaged inverse Fermi velocities (7). This holds especially for NbP. The general result breaks the physical picture that 3D Weyl nodes generates finite values of the interband contributions to Im*ε*_*jj*_(0) for vanishing photon energies and occupations. Details on the breaking of this picture at the 24 Weyl nodes will be further discussed below.

#### Interplay of intraband and interband transitions

The finite Fermi energies differences ${\varepsilon }_{{F}_{i}}$ in Table 2 and the modified quantities in expression (6) can be traced back to degenerate electron or hole gases in the linear bands around the asymmetric Weyl nodes with rather small densities of states. They give rise to intraband contributions to the dielectric functions, which we model by Drude terms^37^. Here, we neglect the polarization anisotropy of the electron or hole gases near the Weyl nodes. Then, applying formula (11) and assuming an average broadening parameter Γ_*D*_ for all Weyl nodes, one may introduce an effective isotropic plasma frequency of the carriers in all 24 nodes, ${\omega }_{pD}={[\frac{4\pi {e}^{2}}{m}\sum _{i=1,2}{w}_{i}{n}_{i}]}^{\mathrm{1/2}}$. Because of the fact that for *W*_1_ the bands are flat in *k*_*z*_-direction, the electron densities in this node give the most significant contributions to the intraband part. The effect of the electron (TaAs) and hole (TaP, NbAs, NbP) densities in *W*_2_ Weyl nodes is rather negligible. The sum over the 24 Weyl nodes leads to effective plasma frequencies *ħω*_*pD*_ = 41.7 (TaAs), 171.7 (TaP), 74.1 (NbAs), and 148.7 meV (NbP). These values are, of course, slightly smaller than those, 70, 222, 185, and 649 meV, obtained with the approximate electron/hole densities in Table 2 computed assuming linear bands (2). The larger experimental value of 608 meV for TaAs^21^ may indicate additional free carriers due to impurities and defects in the investigated samples, but may also refer to free carriers existing near trivial points in the BZ, which are not counted in *ω*_*pD*_.

**Figure 6:**
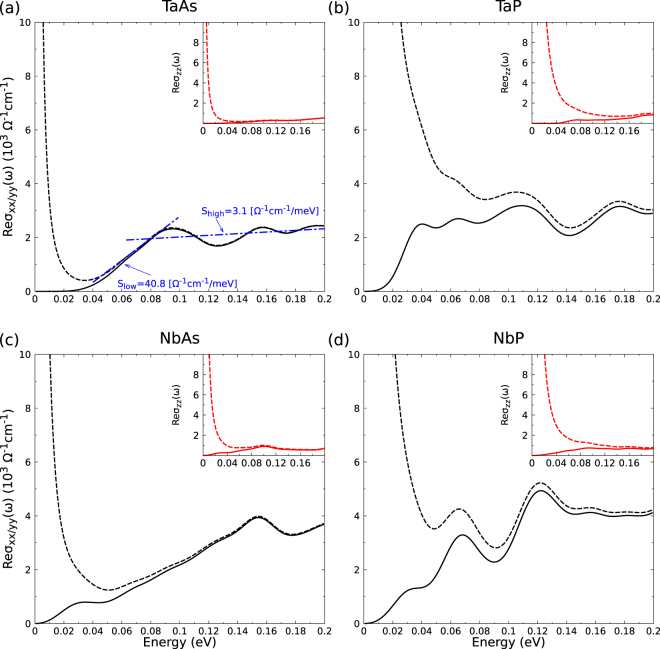
Real part of the optical conductivity versus photon energy for light polarization perpendicular to tetragonal axis (*j* = *x*, *y*), i.e., normal incidence; parallel to the tetragonal axis (*j* = *z*) in the insets. Solid lines: only interband contributions, dashed lines: including Drude terms. For TaAs linear fits guide the eyes in the low and higher energy region.

**Figure 7:**
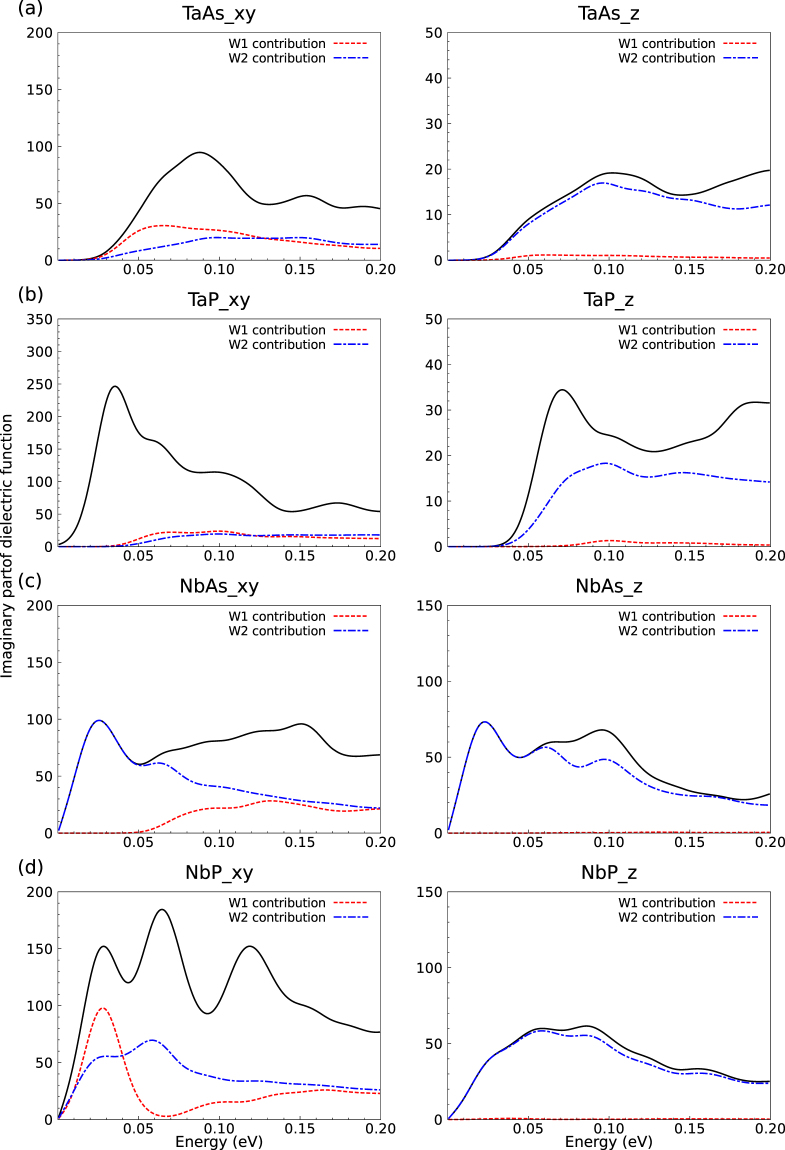
Imaginary part of the dielectric function with the explicit contribution of the W1 and W2 Weyl nodes (Total: black line; *W*1: red dashed line, and *W*2: blue dot-dashed line) for (**a**) TaAs, (**b**) TaP, (**c**) NbAs, and (**d**) NbP. The total spectrum contains *W*_1_ and *W*_2_ contributions as well as those of the trivial points.

For comparison with the measurements for TaAs^21^, we combine the effective Drude term with the interband dielectric function to the optical conductivity *σ*_*jj*_(*ω*). For the real part, assuming only one effective Drude term for the 24 Weyl nodes in the BZ, we find

8 $${\rm{Re}}\,{\sigma }_{jj}(\omega )=\frac{1}{4\pi }\,\{{{\rm{\Gamma }}}_{D}\frac{{\omega }_{pD}^{2}}{{\omega }^{2}+{{\rm{\Gamma }}}_{D}^{2}}+\omega {\rm{Im}}\,{\varepsilon }_{jj}(\omega )\},$$

where the effective scattering time 1/Γ_*D*_ in all Weyl nodes characterizes the carriers mobility. Results for the optical conductivity with and without the Drude effects are plotted in Fig. 6 in the limit of small photon energies. Since the reciprocal scattering time Γ_*D*_ is difficult to compute within an electronic structure code, we apply the parameter *ħ*Γ_*D*_ = 1.46 meV measured for TaAs at 5 K^21^ for both arsenides. In Fig. 6 we focus on normal incidence along the tetragonal axis, i.e., *j* = *x*, *y*. Results for the polarization along the tetragonal axis, i.e. *j* = *z*, are displayed in the insets. Interband and intraband contributions are plotted. The interband contributions are decomposed in Fig. 7 into the contributions around Weyl nodes of the two kinds *W*_1_ and *W*_2_. The total spectra contain also all trivial points in the BZ. These interband contributions calculated with a high **k**-point density show onsets, which in general more or less deviate from the values 2$|{\varepsilon }_{{F}_{i}}|$ for symmetric cones but have to be replaced by 2$|{\varepsilon }_{{F}_{i}}^{\ast }|$ for asymmetric Weyl nodes *W*_*i*_. The consequences for the far-IR absorption spectra in Fig. 6 will be discussed below.

Neglecting the effects of the trivial points, the expected behavior of the optical conductivity may be described in terms of the linear-band approximation. The pure interband contributions of linear bands should exhibit an almost linear slope, at least above the onset values of $2|{\varepsilon }_{{F}_{i}}^{\ast }|$ (*i* = 1, 2), which are in general lower than 2$|{\varepsilon }_{{F}_{i}}|$. Using relations (13) and (6) we obtain

9 $${\rm{Re}}\,{\sigma }_{jj}(\omega )=\frac{{e}^{2}}{h}\omega \frac{1}{24}\,\sum _{i=1,2}\,{w}_{i}{F}_{i}\theta (\hslash \omega -\mathrm{2|}{\varepsilon }_{{F}_{i}}^{\ast }|)\frac{1}{{\bar{v}}_{{F}_{j}}^{i}}\mathrm{.}$$

The reciprocal von Klitzing constant *h*/*e*^2^ = 2.581 × 10^4^ Ω (in SI units) leads to the unit Ω^−1^ cm^−1^ of the optical conductivity. Expression (9) shows that the interband contributions are expected for photon energies above the minimum value of 2$|{\varepsilon }_{{F}_{i}}^{\ast }|$. Because of the asymmetry and the tilting of the cones, the Weyl node character of the lowest onset is not clear. Neglecting the tilting it holds ${\varepsilon }_{{F}_{i}}^{\ast }={\varepsilon }_{{F}_{i}}$ and a linear increase happens due to the occupation-allowed transitions around the 16 *W*_2_ Weyl nodes from $\hslash \omega =2|{\varepsilon }_{{F}_{2}}|$. With the Fermi velocities of Table 3 and using (9), the slope parameter *S*_*low*_ = *dReσ*_*jj*_(*ω*)/*dħω* in units of (Ω · cm · meV)^−1^ becomes *S*_*low*_ = 8.1 (8.2) for TaAs, 8.9 (8.8) for TaP, 10.6 (10.3) for NbAs, and 12.3 (8.8) for NbP for light polarization perpendicular (parallel) to the tetragonal axis. Above $\hslash \omega  > 2|{\varepsilon }_{{F}_{1}}|$ also the *W*_1_ nodes should contribute. The linear slope further increases to values as high as *S*_*high*_ = 11.9 (69.9) for TaAs, 12.4 (70.5) for TaP, 15.0 (133.6) for NbAs, and 16.2 (419.8) for NbP due to contributions from both kinds of Weyl nodes *W*_2_ and *W*_1_.

These expectations based on symmetric and untilted cones are, however, not in agreement with IR interband spectra calculated *ab*-*initio* with a refined **k**-point set as shown in Fig. 6. Their lineshapes and their onsets are modified by the asymmetry and tilting of the Weyl fermions and the subsequent modified onsets of the interband transitions as well as contributions of trivial points. These effects are illustrated in Figs. 3 and 7. The larger contribution of the trivial points appears especially for TaP and NbP *x* and *y* polarizations. The vertical arrows in Fig. 3 between lower (−) and upper (+) Weyl bands show the lowest allowed optical interband transitions in agreement with the band occupation. Additional arrows indicate transitions between Weyl and close non-topological bands, whose oscillator strengths are however smaller. Pronounced differences to the picture of symmetric Weyl fermions are the lower onsets of the order of *ħω* = 0.03 eV of the *W*_1_ nodes in the spectra of TaAs and TaP (Fig. 7a,b; *x* and *y* polarization), in contrast to the fact that $|{\varepsilon }_{{F}_{2}}|$ is much smaller that $|{\varepsilon }_{{F}_{1}}|$ as given in Table 2 and the onset of $2|{\varepsilon }_{{F}_{1}}|$ = 0.052 eV or 0.110 eV expected for symmetric Weyl fermions. The move of the onsets to lower energies is indicated by the lengths of the arrows in Fig. 3a,b (left panels). For the Nb compounds in Fig. 7c,d, the influence of the *W*_1_ nodes is generally suppressed with the exception of *x* and *y* polarization in NbP. The onsets of the *W*_2_ contributions are shifted to vanishing photon energies. This is in agreement with $|{\varepsilon }_{{F}_{2}}|$ in Table 3 for NbAs but occurs for reasons which are illustrated in Fig. 3c,d. In contrast to the expectations, for *x* and *y* polarizations the IR spectra of TaP and NbAs are stronlgy influenced at the onsets. For NbP this happens for slightly higher photon energies. These facts make the optical observation of Weyl fermions difficult for light polarization perpendicular to the tetragonal axis. However, Fig. 7, indicates that for *z*-polarization *W*_2_ Weyl fermions dominate the interband spectrum. They are observable in the optical conductivity in Fig. 6. This fact should be taken into consideration for future optical studies of Weyl fermions in the monopnictides. The *z* polarization is favored to investigate the *W*_2_ fermions for all monopnictides (but TaP), whereas the *x* and *y* polarization makes such optical studies meaningful mainly for NbAs.

The striking features of the low-temperature optical conductivity of TaAs and (probably) *x* or *y* polarization in Fig. 4 of ref.^21^ are two frequency-linear components with distinct slopes *S*_*low*_ = 56.7 (Ω · cm · meV)^−1^ and *S*_*high*_ = 4.1 (Ω · cm · meV)^−1^ above the Drude contribution which seem to be hallmarks of a TWS as indicated by formula (9). The *ab*-*initio* calculations presented in Fig. 6a and combined with the Drude term are in qualitative agreement with the experimental data. This holds especially for the two almost linear components in the spectrum with slope parameters of the same order of magnitude as observed experimentally. However, the smaller slope-parameter *S*_*high*_ for the high-energy region already indicates deviations from the simple Weyl fermion picture. According to formula (9) it should be larger, and not smaller, than the slope parameter *S*_*low*_ of the linear frequency component for lower energies. The decomposition in Fig. 7a suggests that the high-energy spectrum is determined by *W*_1_ and *W*_2_ Weyl fermions, but also by carriers from trivial points in the BZ. Moreover, the details of the band structure in Fig. 3 induce some deviations from the strict linear frequency behavior. Similar arguments hold for the onset. There is also a noticible discrepancy between theory and experiment: the onset and the kink at about 30 meV in the measured spectrum are redshifted with respect to the calculated spectrum. This fact may be related to other carrier concentrations and the appearence of phonons around 32 meV in the real TaAs sample. In addition, the Kramers-Kronig transformation of the measured reflectivity spectrum into the real part of the optical conductivity is based on the assumptions that the reflectivity is almost a constant until 12.5 eV and decreases as *ω*^−4^. For this reason, we present in the Supplementary Material (SM) reflectivity spectra calculated for normal incidence using the Fresnel formula and the dielectric functions plotted in Figs. 4 and 5. For TaAs and TaP we show that for high energies (in the range 18–30 eV) a strong reflectivity appears due to transitions from Ta4*f* bands into the lowest conduction band states.

## Summary and Conclusions

The atomic geometries, band structures and dielectric functions for bct TaAs, TaP, NbAs and NbP have been calculated in the framework of DFT including SOI. The resulting lattice and internal-cell parameters agree well with results of measurements and similar calculations. The accompanying band structures exhibit small energetic overlaps at 24 Weyl nodes in the BZ with the Fermi level, thereby explaining the semi-metallic character of the compounds. The Weyl nodes can be divided in four pairs of *W*_1_ and eight pairs of *W*_2_ characters. The lack of inversion symmetry, the linearity of the bands near the Fermi energy, their non-degeneracy, and the pairwise arrangement of the nodes in the BZ illustrate the character of the compounds as topological Weyl semimetals with low-density electron (hole) gases around *W*_1_ (*W*_2_) nodes. With solely electron gases at *W*_1_ and *W*_2_ Weyl points TaAs represents an exception. For the fermions near the Weyl nodes in one pair we have illustrated the influence of tilting and strong anisotropic deformation of the Dirac cones for small energy intervals around the Fermi level in the band structures.

The interband and intraband optical transitions near the Weyl nodes rule the lineshape of the optical spectra, e.g. dielectric function and optical conductivity, in the low-frequency region. On average, the imaginary parts of the interband dielectric function give rise to lineshapes ruled by the band structure details and the band occupation. In addition, they depend strongly on the light polarization. The rough linearity of the real part of the optical conductivity in two frequency intervals is due to the low-density electron and hole gases around *W*_1_ and *W*_2_ nodes and is in qualitative agreement with measurements for TaAs. However, the findings need a refined interpretation due to the anisotropy and tilting of the Weyl nodes as well as the appearence of trivial points. The almost two frequency-linear experimental contribution to the optical conductivities for TaAs and the simplified isotropic Weyl fermion picture seem to represent hallmarks of TWSs due their linear bands near the Weyl nodes. Nevertheless, the details of the bands near the pairs of Weyl nodes, such as high anisotropy, tilt and position with respect to the Fermi level, modify this simplified picture. The asymmetry and the tilting of the Weyl fermions have to be taken into account and be combined with details of the band structure around the Weyl node at slightly higher energies. Moreover, the occurrence of trivial points with carriers with non-linear dispersion plays a role. In particular, for NbAs (*x*, *y* and *z* polarization) but also for NbP and TaAs (*z* polarization), we suggest experiments to attack optically the properties of *W*_2_ Weyl fermions.

## Methods

For the structural, electronic and optical properties of the monopnictides we apply the density functional theory (DFT) as implemented in the code Quantum Espresso Package^38^. Exchange and correlation (XC) are treated within the generalized gradient approximation (GGA) of Perdew, Burke and Ernzerhof (PBE)^39^. The electron-ion interaction is described by norm-conserving, fully relativistic pseudopotentials for the *s* and *p* valence electrons and for the partially filled semicore *d* states of Nb and Ta. Other shallow core states such as Ta4*f* ones are taken into account. More in detail, we study pseudoatoms with electronic configurations 4*s*^2^4*p*^6^4*d*^3^5*s*^2^ (Nb), 4*f*^14^5*s*^2^5*p*^6^5*d*^3^6*s*^2^ (Ta), 4*s*^2^4*p*^3^ (As), and 3*s*^2^3*p*^3^ (P). Despite the higher numerical effort for the structural calculations, the norm conservation is requested to compute the optical matrix elements in a reasonable manner. The cut-off radii for each element and orbital state are chosen in such a way that atomic all-electron and pseudowave functions agree in a suitable manner. The validity of each pseudopotential has been tested in several respects, in particular, by comparing the all-electron and pseudo wave functions and their logarithmic derivatives to guarantee the absence of ghost states^40^. Besides the mass and Darwin terms the inclusion of SOI has been done by, first, solving the radial Dirac equation for each isolated atom and, second, by reducing the four-component Dirac spinors to two-component Pauli spinors in order to generate pseudopotentials with two-component projectors^41,42^.

The BZ of the bct crystals is sampled by a uniform grid of 21 × 21 × 7 Monkhorst-Pack *k* points^43^. Besides the **k**-point density the convergence of the total energy *E*_tot_ of the crystals is also determined by the cutoff energy of the plane waves. When we allow a change of *E*_tot_ by less than 0.0005 Ry for an increase of *E*_cut_ by 10 Ry, the convergence tests indicate that a parameter *E*_cut_ = 100 Ry is sufficient.

In order to compute the electronic structures, in principle, quasiparticle corrections^34^ have to be added. However, there is a tendency for compensation of quasiparticle blueshifts and excitonic redshifts in absorption spectra to account for the excitation aspect. Moreover, in real semimetals with free electron and hole carriers the many-body effects are reduced due to the increased screening. Finally, near the Fermi energy, the quasiparticle corrections should be negligible. Therefore, the electronic structures are computed as Kohn-Sham eigenvalues and eigenfunctions of the DFT.

The frequency-dependent dielectric tensor *ε*_*ij*_(*ω*) is calculated within the independent-particle approach^44^. The imaginary part of the diagonal elements (*j* = *x*, *y*, *z*) is given as^34,37^

10 $$\begin{array}{rcl}{\rm{Im}}{\varepsilon }_{jj}^{{\rm{inter}}}(\omega ) & = & {(\frac{2\pi e}{m\omega })}^{2}\frac{1}{V}\,\sum _{{\bf{k}}}\,\sum _{c,v}\,[f({\varepsilon }_{v}({\bf{k}}))-f({\varepsilon }_{c}({\bf{k}}))]\\  &  & \times {|\langle c{\bf{k}}|{p}_{j}|v{\bf{k}}\rangle |}^{2}\delta ({\varepsilon }_{c}({\bf{k}})-{\varepsilon }_{v}({\bf{k}})-\hslash \omega )\end{array}$$

with *V* as the crystal volume. Here, only the interband contributions with transitions between valence (|*v***k**〉) and conduction (|*c***k**〉) Bloch states (described by Pauli spinors) with eigenvalues *ε*_*c*_(**k**) and *ε*_*v*_(**k**) and Fermi occupation numbers *f*(*ε*) are displayed. *p*_*j*_ represents a Cartesian component of the momentum operator. The intraband contributions are modeled by isotropic Drude terms

11 $${\varepsilon }_{jj}^{{\rm{intra}}}(\omega )=1-\sum _{i=1,2}\,{w}_{i}\frac{{\omega }_{pi}^{{\ast }^{2}}}{\omega (\omega +i{{\rm{\Gamma }}}_{i})}$$

for all *W*_*i*_ (*i* = 1, 2) Weyl nodes with numbers *w*_1_ = 8 and *w*_2_ = 16 and effective plasma frequencies

12 $${\omega }_{{p}_{i}}^{{\ast }^{2}}=\frac{4\pi {e}^{2}}{m}{n}_{i},$$

where the electron or hole density *n*_*i*_ at a Weyl node of type *i* = 1, 2 in the band *ν* crossed by the Fermi level

$${n}_{i}={\int }_{0}^{|{\varepsilon }_{Fi}|}\,d\varepsilon {D}_{i}(\varepsilon )$$

is related to the density of states around a Weyl node ${{\bf{k}}}_{{W}_{i}}$, i.e., for small energies,

$${D}_{i}(\varepsilon )={\int }_{({{\bf{k}}}_{{W}_{i}})}\,\frac{{d}^{3}{\bf{k}}}{{\mathrm{(2}\pi )}^{3}}\delta (\varepsilon -{\varepsilon }_{\nu }({\bf{k}}\mathrm{)).}$$

The sign of the energy is accounted appropriately for the partially filled (with electrons or holes) conduction or valence band *ν* around a Weyl node ${{\bf{k}}}_{{W}_{i}}$. The reciprocal plasmon lifetimes are denoted by Γ_*i*_ in eq. (11).

The real part of the dielectric function (10) follows via the Kramers-Kronig relation^34,37^. The dielectric function is related to the optical conductivity by

13 $${\sigma }_{jj}(\omega )=\frac{\omega }{4\pi i}{\varepsilon }_{jj}(\omega \mathrm{).}$$

## Electronic supplementary material

Supplemental material

## References

[1] Young S (2012). Dirac Semimetal in Three Dimensions. Phys. Rev. Lett..

[2] Borisenko S (2014). Experimental realization of a three-dimensional Dirac semimetal. Phys. Rev. Lett..

[3] Liu Z (2014). Discovery of a three-dimensional topological Dirac semimetal, Na_3_Bi. Sci..

[4] Xu G, Weng H, Wang Z, Dai X, Fang Z (2011). Chern semimetal and the quantized anomalous Hall effect in HgCr_2_Se_4_. Phys. Rev. Lett..

[5] Kane CL, Mele EJ (2005). Quantum spin Hall effect in graphene. Phys. Rev. Lett..

[6] Matthes L, Pulci O, Bechstedt F (2013). Massive Dirac quasiparticles in the optical absorbance of graphene, silicene, germanene, and tinene. Journal of Physics: Condensed Matter.

[7] Burkov A, Balents L (2011). Weyl semimetal in a topological insulator multilayer. Phys. Rev. Lett..

[8] Burkov A, Hook M, Balents L (2011). Topological nodal semimetals. Phys. Rev. B.

[9] Wan X, Turner AM, Vishwanath A, Savrasov SY (2011). Topological semimetal and Fermi-arc surface states in the electronic structure of Pyrochlore Iridates. Phys. Rev. B.

[10] Huang S-M (2015). A Weyl fermion semimetal with surface Fermi arcs in the transition metal monopnictide TaAs class. Nat. communications.

[11] Hasan, M., Xu, S. & Bian, G. Corrigendum: Topological insulators, topological superconductors and Weyl fermion semimetals: discoveries, perspectives and outlooks. *Phys*. *Scr* 014001 (2015).

[12] Lv B (2015). Experimental discovery of Weyl semimetal TaAs. Phys. Rev. X.

[13] Souma S (2016). Direct observation of nonequivalent Fermi-arc states of opposite surfaces in the noncentrosymmetric Weyl semimetal NbP. Phys. Rev. B.

[14] Xu S-Y (2015). Discovery of a Weyl fermion semimetal and topological Fermi arcs. Sci..

[15] Xu S-Y (2015). Experimental discovery of a topological Weyl semimetal state in TaP. Sci. Adv..

[16] Belopolski I (2016). Criteria for directly detecting topological Fermi arcs in Weyl semimetals. Phys. Rev. Lett..

[17] Xu S-Y (2016). Spin polarization and texture of the Fermi arcs in the Weyl fermion semimetal TaAs. Phys. Rev. Lett..

[18] Zhang C-L (2016). Signatures of the Adler–Bell–Jackiw chiral anomaly in a Weyl fermion semimetal. Nat. Commun..

[19] Klotz J (2016). Quantum oscillations and the Fermi surface topology of the Weyl semimetal NbP. Phys. Rev. B.

[20] Wang Z (2016). Helicity-protected ultrahigh mobility Weyl fermions in NbP. Phys. Rev. B.

[21] Xu B (2016). Optical spectroscopy of the Weyl semimetal TaAs. Phys. Rev. B.

[22] Hills RD, Kusmartseva A, Kusmartsev F (2017). Current-voltage characteristics of Weyl semimetal semiconducting devices, Veselago lenses, and hyperbolic Dirac phase. Phys. Rev. B.

[23] Grushin AG, Bardarson JH (2017). How to Make Devices with Weyl Materials. Phys..

[24] Castelvecchi D (2017). The strange topology that is reshaping physics. Nat. News.

[25] Weng H, Fang C, Fang Z, Bernevig BA, Dai X (2015). Weyl semimetal phase in noncentrosymmetric transition-metal monophosphides. Phys. Rev. X.

[26] Lee C-C (2015). Fermi surface interconnectivity and topology in Weyl fermion semimetals TaAs, TaP, NbAs, and NbP. Phys. Rev. B.

[27] Buckeridge J, Jevdokimovs D, Catlow C, Sokol A (2016). Bulk electronic, elastic, structural, and dielectric properties of the Weyl semimetal TaAs. Phys. Rev. B.

[28] Chang G (2016). Signatures of Fermi arcs in the quasiparticle interferences of the Weyl semimetals TaAs and NbP. Phys. Rev. Lett..

[29] Xu N (2017). Distinct evolutions of Weyl fermion quasiparticles and Fermi arcs with bulk band topology in Weyl semimetals. Phys. Rev. Lett..

[30] Furuseth S, Selte K, Kjekshus A (1965). On the Arsenides and Antimonides of Tantalum. Acta Chem. Scand..

[31] Martienssen W, Warlimont H (2005). Springer handbook of condensed matter and materials.

[32] Kittel, C. *Introduction to solid state physics* (Wiley, 2005).

[33] de Carvalho LC, Schleife A, Bechstedt F (2011). Influence of exchange and correlation on structural and electronic properties of AlN, GaN, and InN polytypes. Phys. Rev. B.

[34] Bechstedt F (2015). Many-Body Approach to Electronic Excitations. Concepts and Applications.

[35] Mosca Conte A, Pulci O, Bechstedt F (2017). Electronic and optical properties of topological semimetal Cd_3_As_2_. Sci. Reports.

[36] Matthes L, Gori P, Pulci O, Bechstedt F (2013). Universal infrared absorbance of two-dimensional honeycomb group-IV crystals. Phys. Rev. B.

[37] Grosso G, Pastori Parravicini G (2000). Solid State Physics.

[38] Giannozzi, P. *et al*. QUANTUM ESPRESSO: a modular and open-source software project for quantum simulations of materials. *J*. *Physics*: *Condens*. *Matter* **21**, 395502 (19pp), http://www.quantum-espresso.org (2009). 10.1088/0953-8984/21/39/395502 21832390

[39] Perdew JP, Burke K, Ernzerhof M (1996). Generalized gradient approximation made simple. Phys. RReview Lett..

[40] Gonze X, Käckell P, Scheffler M (1990). Ghost states for separable, norm-conserving, ab initio pseudopotentials. Phys. Rev. B.

[41] Dal Corso A, Conte AM (2005). Spin-orbit coupling with ultrasoft pseudopotentials: Application to Au and Pt. Phys. Rev. B.

[42] Conte AM, Fabris S, Baroni S (2008). Properties of Pt-supported Co nanomagnets from relativistic density functional theory calculations. Phys. Rev. B.

[43] Monkhorst HJ, Pack JD (1976). Special points for Brillouin-zone integrations. Phys. Rev. B.

[44] Adolph B, Gavrilenko V, Tenelsen K, Bechstedt F, Del Sole R (1996). Nonlocality and many-body effects in the optical properties of semiconductors. Phys. Rev. B.

