# Supplementary material for: Validity of Weyl fermion picture for transition metals monopnictides TaAs, TaP, NbAs, and NbP from ab initio studies
Source: Sci Rep. 2018 Feb 23;8:3534. doi: 10.1038/s41598-018-21465-z (PMC5824971; doi:10.1038/s41598-018-21465-z)
Supplement: Supplementary file 1 — Supplemental material [file 41598_2018_21465_MOESM1_ESM.pdf]

Supplemental material for : "Validity of Weyl fermion picture for transition metals monopnictides TaAs, TaP, NbAs and NbP from *ab initio* studies"

Davide Grassano[1], Olivia Pulci[1] Adriano Mosca Conte[2] and Friedhelm Bechstedt[3]
[1] *Dipartimento di Fisica, Università di Roma Tor Vergata, Via della Ricerca Scientifica 1, I-00133 Rome, Italy*
[2] *Mediterranean Institute of Fundamental Physics (MIFP), Via Appia Nuova 31, I-00040 Marino, Rome, Italy*
[3] *Institut für Festkörpertheorie und -optik, Friedrich-Schiller-Universität, Max-Wien-Platz 1, 07743 Jena, Germany*
(Dated: January 19, 2018)

The nodal lines, to which the pairs of Weyl nodes in Fig.1 belong, have been found by using a dense (20000) mesh of k-points on the mirror plane

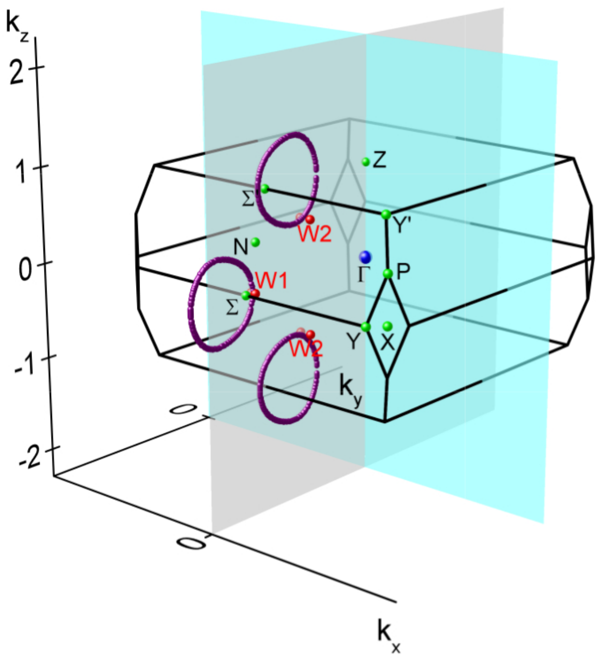

FIG. S1: Plot of the nodal lines in the BZ.

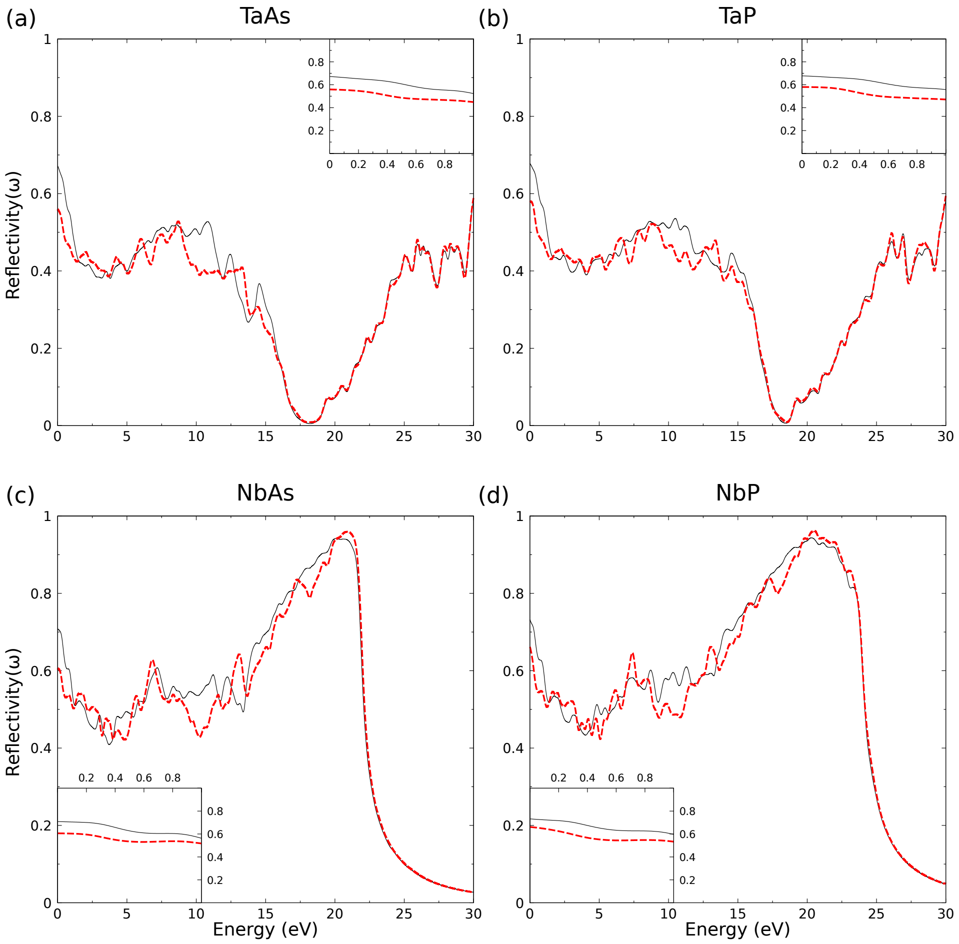

FIG. S2: Reflectivity of (a) TaAs, (b) TaP, (c) NbAs, and (d) NbP calculated for light polarization perpendicular (black solid lines) and parallel (red solid lines) to the tetragonal axis. The insets show the same data in a smaller energy range.
